# *GJA8*-associated developmental eye disorders: a new multicentre study highlights mutational hotspots and genotype-phenotype correlations

**DOI:** 10.1038/s41431-025-01843-8

**Published:** 2025-04-30

**Authors:** Solomon S. Merepa, Linda M. Reis, Alejandra Damián, Tanya Bardakjian, Adele Schneider, María Jose Trujillo-Tiebas, Carmen Ayuso, Laura Cortázar Galarza, Raquel Saez Villaverde, Nelmar Valentina Ortiz-Cabrera, Dorine A. Bax, Richard Holt, Fabiola Ceroni, Patrick Edery, Maude Grelet, Florence Riccardi, Lauriane Maillard, Deborah Costakos, Julie Plaisancié, Nicolas Chassaing, Marta Corton, Elena V. Semina, Nicola K. Ragge

**Affiliations:** 1 Faculty of Health, Science and Technology, School of Biological and Medical Sciences, Oxford Brookes University, Headington Campus, Gipsy Lane, Oxford, UK; 2 Department of Ophthalmology and Visual Sciences, Medical College of Wisconsin, Milwaukee, WI USA; 3 Department of Genetics & Genomics, Instituto de Investigación Sanitaria-Fundación Jiménez Díaz University Hospital - Universidad Autónoma de Madrid (IIS-FJD, UAM), Madrid, Spain; 4 U704 Centro de Investigación Biomédica en Red de Enfermedades Raras (CIBERER), Madrid, Spain; 5 Einstein Medical Center Philadelphia, Philadelphia, PA USA; 6 Wills Eye Hospital, Philadelphia, PA USA; 7 Hospital Universitario Donostia, San Sebastián, Spain; 8 Department of Genetics, Hospital Infantil Universitario Niño Jesús, Madrid, Spain; 9 Université Claude Bernard Lyon 1, INSERM, CNRS, Centre de Recherche en Neurosciences de Lyon CRNL U1028 UMR5292, Genetics of Neurodevelopment Team, Bron, France; 10 Department of Genetics, Clinical Genetics Unit, Centre de Référence Maladies Rares des Anomalies du Développement, Hospices Civils de Lyon, Université Claude Bernard Lyon 1, Bron, France; 11 Centre Hospitalier Intercommunal de Toulon- La Seyne sur mer, Service de Génétique Médicale, Toulon, France; 12 Service d’Opthalmologie, Hôpital Purpan, CHU Toulouse, Toulouse, France; 13 Laboratoire de Référence (LBMR) des anomalies malformatives de l’œil, Institut Fédératif de Biologie (IFB), CHU Toulouse, Toulouse, France; 14 Centre de Référence des Affections Rares en Génétique Ophtalmologique CARGO, Site Constitutif, CHU Toulouse, Toulouse, France; 15 Molecular, Cellular and Developmental Biology Unit (MCD), Centre de Biologie Intégrative (CBI), Université de Toulouse, CNRS, UPS, Toulouse, France; 16 Department of Pediatrics, Medical College of Wisconsin, Milwaukee, WI USA; 17 West Midlands Regional Clinical Genetics Service and Birmingham Health Partners, Birmingham Women’s and Children’s Foundation Trust, Birmingham, UK

**Keywords:** Development, Genetics research

## Abstract

Variants in *gap junction protein alpha 8* (*GJA8*), the gene encoding connexin 50 (Cx50), are primarily associated with developmental cataract, although some are associated with severe structural eye anomalies, such as aphakia (absent lens), microphthalmia (small eyes), and sclerocornea. To further define the relationship of *GJA8* variants to ocular developmental disorders, we screened four large international cohorts with structural eye anomalies, including anophthalmia, microphthalmia, and coloboma (AMC) or cataracts. We identified 15 new families carrying 14 different heterozygous *GJA8* variants (12 missense variants and two 1q21 microdeletions). The missense variants comprised 10 previously reported alterations in cases with eye anomalies [p.(Gly22Ser), p.(Val44Met), p.(Asp67Gly), p.(Arg76Cys), p.(Pro88Leu), p.(Gly94Glu), p.(Gly94Arg), p.(His98Arg), p.(Pro189Ser), and p.(Arg198Trp)] and two not yet linked with disease [p.(Thr39Met) and p.(Tyr66Asp)]. Their associated phenotypes ranged from isolated cataracts to a combination of microphthalmia and cataract with/without sclerocornea. Our study confirms *GJA8* variants as an important source of genetic diagnoses for families with structural eye anomalies in addition to cataract and highlights specific mutational hotspots. Furthermore, we confirm an important genotype-phenotype correlation between sclerocornea and the p.(Gly94Arg) variant, and detail intra- and inter-familial phenotypic variability, which is important for clinical assessment and genetic counselling.

## Introduction

Developmental eye anomalies, such as anophthalmia (absent eye), microphthalmia (small eye) and coloboma (optic fissure closure defects) (AMC), affect approximately 11.9 per 100,000 live births [1]. AMC conditions are responsible for around 15–21% of childhood blindness worldwide, and together with congenital retinal and optic nerve disorders, are estimated to account for up to 40-50% in some countries [2, 3]. They can be either isolated or occur with other ocular anomalies (including cataract and anterior segment dysgenesis) and/or extraocular features [4]. The genetic basis of these anomalies is highly heterogenous, reflecting the complexity of the genes and pathways involved in human eye development. Currently, 147 genes/loci are considered diagnostic on the Genomics England structural eye disease genetic testing panel (PanelApp; https://panelapp.genomicsengland.co.uk/panels/509/; Version 4.1).

The role of *GJA8* (*gap junction protein alpha 8*) in mammalian eye development and congenital eye anomalies has been previously explored by animal models [5, 6]. *GJA8* encodes connexin 50 (Cx50), a transmembrane protein involved in the formation of gap junctions essential for the maintenance of mammalian lens transparency [7]. Like other connexins, Cx50 consists of four conserved transmembrane alpha helices (TM1, 2, 3, and 4) joined by two extracellular loops (ECL1 and ECL2) and one cytoplasmic loop, and flanked by cytoplasmic N- and C-terminal domains. Connexins form hexameric complexes (connexons) that either function as hemi-channels or dock with counterparts on adjoining cells to form intercellular channels, allowing the exchange of small molecules. Cx50 is also able to form heteromeric gap junction channels with other connexins, including Cx46 (GJA3), enabling gap junction channels to have different properties depending on their specific connexin composition [8]. Beyond its channel-forming roles, some Cx50 protein domains are involved in other essential processes, including regulating cell adhesion, lens differentiation, and expression levels of cell adhesion molecules, such as N-cadherin and β-catenin [9].

Individuals with pathogenic *GJA8* variants typically display lens anomalies, most commonly congenital cataract [8, 10], and, more rarely, aphakia (absent lens) [11–13]. However, other developmental eye anomalies sometimes occur, including microphthalmia, microcornea, coloboma and sclerocornea, typically co-occurring with cataract [11, 12, 14]. This indicates that, beyond its specific role in lens development, *GJA8* is also more widely involved in ocular growth and development [5, 11]. To date, the majority of pathogenic *GJA8* variants reported are dominantly inherited or de novo heterozygous missense changes, although a few cases have homozygous and compound heterozygous variants [15, 16]. Some individuals with cataract and other developmental eye disorders carry heterozygous 1q21 microdeletions involving *GJA8*, but with incomplete penetrance, creating uncertainty about the contribution of these 1q21 microdeletions to disease [11].

While the involvement of *GJA8* variants in structural eye anomalies is well established, their prevalence and genotype-phenotype correlations are less clear. To delineate this further, we screened four large international cohorts of families with developmental eye disorders for single nucleotide and copy number variants (SNVs/CNVs) affecting *GJA8*. Here, we describe 13 new families with 12 missense variants and two with 1q21 microdeletions. We determine the range of phenotypic features associated with these variants and emerging genotype-phenotype correlations.

## Materials and methods

The cohorts included families with developmental eye anomalies recruited from the UK (n = 425, National Genetics of Eye and Brain anomalies Study UK [Cambridge East Ethics Committee (04/Q0104/129)]) (families 7 and 8), France (n = 445, via diagnostic testing; under French Ethics and Regulatory Law [public health code] specific ethics approval is not required for this study) (families 1, 11 and 12), Spain (n = 462, Genetics of Congenital Ocular Disorders study, Fundación Jimenez Díaz University Hospital [Ethics Research Committee FJD (PIC015-18)]) (families 2, 4, 14 and 15), and the USA (n = 824, Genetic Studies of Human Ocular Disorders, Institutional Review Board of the Medical College of Wisconsin [PRO45954], Children’s Wisconsin [124172], and Einstein Healthcare Network [HN2191]) (families 3, 5, 6, 9, 10 and 13). The entire cohort numbers include some individuals previously screened for *GJA8* variants and reported in previous studies [11, 17], although the cases described herein are new. Consent was obtained from all participants according to the tenets of the Declaration of Helsinki.

*GJA8* variants (reported here according to human Genome Build GRCh38/hg38) were identified using whole genome/exome or clinical exome sequencing (WGS/WES/CES) or customized NGS panels for developmental eye anomalies. CES data were analyzed using the commercial SOPHiA DDM Platform (Sophia Genetics). WGS/WES data were annotated and filtered using commercial software or in-house pipelines, prioritizing rare variants in coding regions or canonical splice sites, predicted pathogenic and consistent with the expected inheritance pattern (see Supplementary Methods for further details). Potentially pathogenic variants in genes implicated in developmental eye disorders were characterized. Individuals with additional systemic features were examined by the appropriate clinical specialists and, where applicable, were tested on additional relevant diagnostic panels for causative variants in genes relevant to the additional features. Functional effects of missense *GJA8* variants were predicted using in silico tools, including SIFT [18], Polyphen-2 [19], CADD [20] and AlphaMissense [21]. Conservation of amino acids across species was determined using GERP++ Rejected Substitutions (RS) score [22] retrieved from the dbNSFP v4.7a database (http://database.liulab.science/dbNSFP). Variants of interest were validated and segregation analyses performed using Sanger sequencing. Variants were classified according to ACGS Best Practice Guidelines for Variant Classification in Rare Disease 2024 [23], a revised version of the ACMG/AMP guidelines [24]. Copy number variants were detected through WES/CES and/or microarray-based comparative genomic hybridization (aCGH).

## Results

**Fig. 1:**
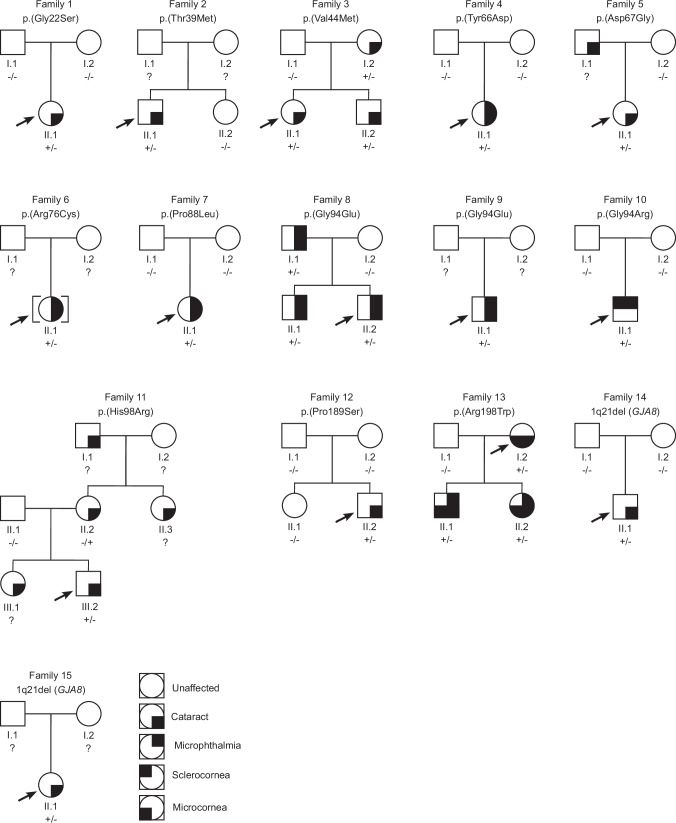
Pedigrees of families reported in the present study. Affected individuals in Families 1-13 carry heterozygous missense *GJA8* variants. Affected individuals in Families 14 and 15 carry heterozygous 1q21 microdeletions affecting *GJA8*. Arrows indicate probands; “?” indicate unknown genotypes.

**Fig. 2:**
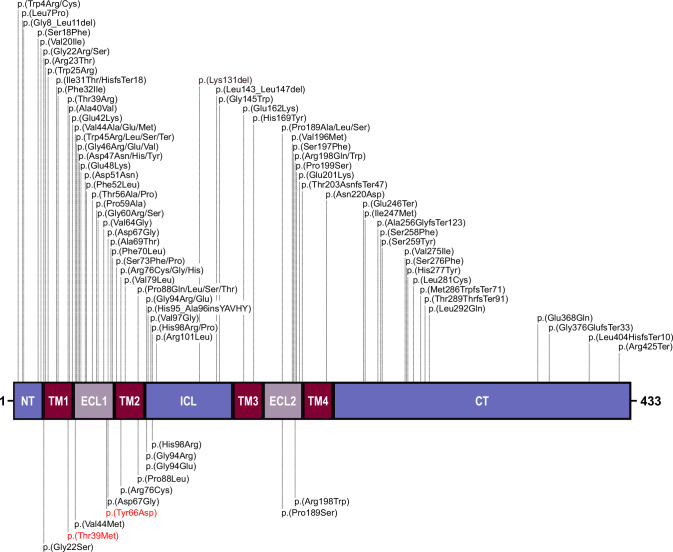
Schematic of the GJA8 protein showing the location of variants associated with structural eye disorders. GJA8 variants are indicated according to their amino acid positions. The functional protein domains are labelled according to Uniprot (entry ID: P48165). Previously published variants associated with developmental eye anomalies are shown above the protein schematic, and missense variants identified in the present study are marked below. The novel variants identified in the present study are indicated in red. NT N-terminal domain, TM transmembrane domain, ECL extracellular loop, ICL intracellular cytoplasmic loop, CT C-terminal domain.

Twenty-two individuals from 15 previously unreported families with developmental eye anomalies carried 12 heterozygous missense *GJA8* variants (13 families) and two heterozygous 1q21 deletions affecting *GJA8* (two families) (Figs. 1 and 2). The 12 heterozygous missense *GJA8* variants were absent or very rare in the gnomAD database (v4.1.0) and comprised ten pathogenic variants, one likely pathogenic and one variant of uncertain significance (VUS). Table 1 provides a summary of the phenotypic features for each family. All SNVs are reported according to NM_005267.5 and NP_005258.2 (Table 2). Cohorts in this study were also screened for variants in other genes implicated in developmental eye anomalies, in addition to panels or genes relevant to other phenotypic features, where applicable. Therefore, unless stated, genetic analysis did not reveal any other variants of relevance in affected individuals.

**Table 1:** Clinical and molecular characteristics of families.

Family (Individual)	Age	Sex	Variant (Genomic coordinate [hg38])	Segregation	Cataracts	Microphthalmia	Other ocular features	Systemic features
1 (II.1)	7m	F	c.64G>A; p.(Gly22Ser) (Chr1:147908019)	De novo	+(B)	–	–	–
2 (II.1)	19y	M	c.116C>T; p.(Thr39Met) (Chr1:147908071)	Unknown	+(B)	–	–	–
3 (I.2)	45y	F	c.130G>A; p.(Val44Met) (Chr1:147908085)	AD	+(B)	–	Glaucoma, nystagmus	–
3 (II.1)	18y	F			+(B)	–	Glaucoma, nystagmus, astigmatism	–
3 (II.2)	13y	M			+(B)	–	Glaucoma	–
4 (II.1)	1y	F	c.196T>G; p.(Tyr66Asp) (Chr1:147908151)	De novo	+(B)	+(B)	Esotropia, nystagmus, amblyopia	–
5 (II.1)	5y	F	c.200A>G; p.(Asp67Gly) (Chr1:147908155)	Unknown	+(B)	–	Nystagmus	–
6 (II.1)	7y	F	c.226C>T; p.(Arg76Cys) (Chr1:147908181)	Unknown	+(B)	+(B)	Nystagmus, esotropia, glaucoma	Hypodontia, microcephaly, sensorineural hearing loss, developmental delay, white matter lesions in brain, dysuria, sacral dimples, poor weight gain
7 (II.1)	3y	F	c.263C>T; p.(Pro88Leu) (Chr1:147908218)	De novo	+(B)	+ (R)	–	Mild incurving on fourth and fifth toes (also present in mother and grandmother)
8 (I.1)	64y	M	c.281G>A; p.(Gly94Glu) (Chr1:147908236)	AD	+(B)	+(B)	Glaucoma	–
8 (II.1)	16y	M			+(B)	+(B)	Glaucoma	–
8 (II.2)	14y	M			+(B)	+(B)	Glaucoma	–
9 (II.1)	3y	M	c.281G>A; p.(Gly94Glu) (Chr1:147908236)	Unknown	+(B)	+(B)	–	Small corpus callosum, lambdoidal craniosynostosis
10 (II.1)	8y	M	c.280G>C; p.(Gly94Arg) (Chr1:147908235)	De novo	Unknown	+(B)	Sclerocornea	Right kidney aplasia
11 (II.2)	45y	F	c.293A>G; p.(His98Arg) (Chr1:147908248)	AD	+(B)	–	Strabismus, nystagmus, mild bilateral intraocular haemorrhage	–
11 (III.2)	25y	M			+(B)	–	Myopia, glaucoma	Congenital deafness, dyslexia, dysorthographia, ADHD, benign infantile epilepsy
12 (II.2)	1y	M	c.565C>T; p.(Pro189Ser) (Chr1:147908520)	De novo	+(B)	–	–	–
13 (I.2)	36y	F	c.592C>T; p.(Arg198Trp) (Chr1:147908547)	AD	+(B)	–	Microcornea, nystagmus, glaucoma	Missing two lateral incisors
13 (II.1)	4y	M			+(B)	+(B)	Microcornea, nystagmus	Mild microcephaly, speech delay
13 (II.2)	2y	F			+(B)	+(B)	Microcornea, nystagmus, esotropia	Mild microcephaly, mild gross motor delay
14 (II.1)	17y	M	1q21 microdeletion (*GJA8*) (Chr1:146020242-147909267)	De novo	+(B)	–	High myopia	–
15 (II.1)	11y	F	1q21 microdeletion (*GJA8*) (Chr1:145516559-149951620)	Unknown	+(B)	–	Left optic nerve drusen at age 7, bilateral central corneal lamellar opacities	–

*AD* autosomal dominant, *B* bilateral, *R* right eye, *L* left eye, *m* month, *y* year, *M* male, *F* female.

**Table 2:** Characterisation of *GJA8* variants identified in this study.

Genomic coordinates (hg38)	cDNA change	Aa change	Domain	Total MAF (gnomAD v4.1.0)	GERP + + _RS	Polyphen-2 HDIV	SIFT	CADD	Alpha-Missense	ACMG/AMP Classification	Previous studies with same variant
Chr1:147908019	c.64G>A	p.(Gly22Ser)	TM1	0	5.03	D (1.0)	D (0.0)	29.7	P (0.86)	Pathogenic (PS1, PS2, PM1, PM2, PP3)	[28, 30, 32]
Chr1:147908071	c.116C>T	p.(Thr39Met)	TM1	6.2E-7	4.9	D (1.0)	D (0.001)	28.8	A (0.63)	Uncertain significance (PM1, PM5, PP3)	N/A
Chr1:147908085	c.130G>A	p.(Val44Met)	ECL1	0	4.9	D (1.0)	D (0.01)	31	P (0.89)	Pathogenic (PS1, PM1, PM2, PP1, PP3)	[31, 29]
Chr1:147908151	c.196T>G	p.(Tyr66Asp)	ECL1	0	5.2	D (1.0)	D (0.0)	29.7	P (0.96)	Pathogenic (PS2, PM1, PM2, PM5, PP3)	N/A
Chr1:147908155	c.200A>G	p.(Asp67Gly)	ECL1	6.2E-7	5.2	D (1.0)	D (0.0)	26.4	P (0.94)	Pathogenic (PS1, PM1, PP3)	[17]
Chr1:147908181	c.226C>T	p.(Arg76Cys)	TM2	0	5.2	D (1.0)	D (0.001)	31	P (0.88)	Likely Pathogenic (PS1, PM1, PM2, PP3)	[17, 30]
Chr1:147908218	c.263C>T	p.(Pro88Leu)	TM2	0	5.2	D (1.0)	D (0.0)	32	P (0.92)	Pathogenic (PS1, PS2, PS3, PM1, PM2, PP3)	[35]
Chr1:147908235	c.280G>C	p.(Gly94Arg)	ICL	0	5.2	D (1.0)	D (0.001)	29.1	P (0.92)	Pathogenic (PS1, PM1, PM2, PM6, PP3)	[11–13, 33]
Chr1:147908236	c.281G>A	p.(Gly94Glu)	ICL	6.2E-7	5.2	D (1.0)	D (0.001)	27.5	P (0.95)	Pathogenic (PS1, PS3, PM1, PP1, PP3)	[12, 14]
Chr1:147908248	c.293A>G	p.(His98Arg)	ICL	0	5.2	D (1.0)	D (0.001)	24.2	A (0.65)	Pathogenic (PS1, PM1, PM2, PP1, PP3)	[32]
Chr1:147908520	c.565C>T	p.(Pro189Ser)	ECL2	0	4.89	D (1.0)	D (0.01)	27.6	P (0.87)	Pathogenic (PS1, PS2, PM1, PM2, PP3)	[27]
Chr1:147908547	c.592C>T	p.(Arg198Trp)	ECL2	0	2.7	D (1.0)	D (0.0)	26.7	P (0.92)	Pathogenic (PS1, PM1, PM2, PP1, PP3)	[30, 36]

cDNA positions refer to NM_005267.5, the protein changes refer to NP_005258.2. Protein domains: *NT* N-terminal domain, *TM* transmembrane domain, *ECL* extracellular loop, *ICL* Intracellular loop, *CT* C-terminal domain. For each variant, total minor allele frequencies from gnomAD (v4.1.0) are reported. Conservation scores (GERP + + _RS) were retrieved from the dbNSFP database v4.7a (accessed in November 2024). GERP++_RS scores range from a minimum of −12.3 to a maximum of 6.17, and positions with scores ≥ 2 are considered conserved. In silico predictions of the functional effects of variants are reported for Polyphen-2, SIFT, CADD and AlphaMissense, retrieved from the dbNSFP database v4.7a (accessed in November 2024) (*D* Damaging, *P* Pathogenic, *A* Ambiguous). Variants were classified as ‘pathogenic’, ‘likely pathogenic’, or ‘uncertain significance’ based on the “ACGS Best Practice Guidelines for Variant Classification in Rare Disease” [23], an updated version of the ACMG/AMP guidelines [24]. *N/A* information not available.

### Family 1

A 7-month-old female (Family 1, II.1) presented with bilateral congenital cataract, and no systemic anomalies. There was no family history of early-onset cataract. She carried a heterozygous pathogenic de novo *GJA8* variant c.64G>A; p.(Gly22Ser).

### Family 2

A 19-year-old male (Family 2, II.1) presented with a history of bilateral congenital cataract, and no additional ocular or systemic anomalies. He carried a heterozygous *GJA8* variant c.116C>T; p.(Thr39Met), classified as a variant of unknown significance. His sister (II.2) did not have congenital eye anomalies, although she presented with subclinical lens opacities at 11 years of age, and tested negative for the variant. Parental DNA samples were unavailable for segregation analysis.

### Family 3

An 18-year-old female (Family 3, II.1) presented with a history of bilateral congenital cataract, glaucoma, nystagmus and astigmatism and no extraocular anomalies. She carried a heterozygous pathogenic *GJA8* variant c.130G>A; p.(Val44Met), which was also detected in her brother (II.2), who presented with bilateral cataract and glaucoma, and her mother (I.2), who also had a history of bilateral cataract, glaucoma and nystagmus.

### Family 4

A 1-year-old female (Family 4, II.1) presented with bilateral congenital cataract, bilateral microphthalmia, esotropia, nystagmus and amblyopia, with no systemic anomalies. There was no family history of eye anomalies. She carried a heterozygous pathogenic de novo *GJA8* variant c.196T>G; p.(Tyr66Asp).

### Family 5

A 5-year-old female (Family 5, II.1) presented with bilateral congenital dense nuclear cataract, nystagmus and no systemic anomalies. She carried a heterozygous pathogenic *GJA8* variant c.200A>G; p.(Asp67Gly), absent in her asymptomatic mother. The proband’s father (I.1) had congenital cataracts with surgery as an infant, but was unavailable for genetic testing.

### Family 6

A 7-year-old female (Family 6, II.1) presented with bilateral congenital nuclear cataract, microphthalmia, infantile glaucoma, nystagmus and esotropia. She also has systemic anomalies including hypodontia, microcephaly, sensorineural hearing loss, developmental delay, white matter lesions in brain, dysuria, sacral dimples and poor weight gain. She carried a heterozygous likely pathogenic *GJA8* variant c.226C>T; p.(Arg76Cys). She also carried a heterozygous variant in *WNT10A* [NM_025216.3: c.682T>A; p.(Phe228Ile), MAF = 0.02 (gnomAD v4.1.0)], associated with an increased risk of tooth agenesis [25] and may account for her hypodontia. The proband was adopted, therefore, the segregation of these variants is unknown.

### Family 7

A 3-year-old female (Family 7, II.1) presented with bilateral congenital cataract and mild microphthalmia in her right eye. She had mild incurving of her fourth and fifth toes, also present in her mother and maternal grandmother, but there was no family history of early onset cataract or microphthalmia. She carried a heterozygous pathogenic de novo *GJA8* variant c.263C>T; p.(Pro88Leu).

### Family 8

A 14-year-old male (Family 8, II.2) presented with bilateral microphthalmia, congenital cataract and glaucoma. His older brother (II.1) and father (I.1) also have bilateral microphthalmia, congenital cataract and glaucoma. All three affected members of the family have no systemic features, and carried a heterozygous pathogenic *GJA8* variant c.281G>A; p.(Gly94Glu).

### Family 9

A 3-year-old boy (Family 9, II.1) presented with bilateral microphthalmia with congenital cataract, small corpus callosum and lambdoidal craniosynostosis. He carried a heterozygous pathogenic *GJA8* variant c.281G>A; p.(Gly94Glu). Parental DNA samples were unavailable for segregation analysis.

### Family 10

An 8-year-old boy (Family 10, II.1) presented with bilateral microphthalmia with bilateral sclerocornea and absent right kidney. The lenses could not be visualised due to the sclerocornea. No further information is available as the proband was unavailable for further examinations. He carried a heterozygous pathogenic de novo *GJA8* variant c.280G>C; p.(Gly94Arg).

### Family 11

A 25-year-old male (Family 11, III.2) presented with a history of bilateral congenital cataract, myopia and glaucoma. Extraocular features included benign infantile epilepsy which resolved after childhood, mild congenital deafness, dyslexia, dysorthographia, and attention deficit hyperactivity disorder. He carried a heterozygous pathogenic *GJA8* variant c.293A>G; p.(His98Arg) inherited from his mother (II.2) who had bilateral congenital cataract, strabismus, nystagmus and mild bilateral intraocular haemorrhage. The proband’s sister (III.1), maternal aunt (II.3) and maternal grandfather (I.1) were reported to be affected with congenital cataract, but their genotypes are unknown. The proband also carried a paternally inherited heterozygous in-frame deletion in *COL11A1* [NM_001854.4: c.3874_3879del; p.(Pro1292_Pro1293del); likely pathogenic (ACMG Class 4)], a gene where heterozygous variants are associated with deafness (DFNA37, OMIM 618533), Marshall syndrome (MRSHS, OMIM 154780) and Stickler syndrome type II (STL2, OMIM 604841). In addition to cataracts, both Marshall and Stickler syndrome can include myopia and sensorineural hearing loss, phenotypes which are also present in the proband’s father (II.1); the *COL11A1* variant could explain the deafness in the proband.

### Family 12

A 1-year-old male (Family 12, II.2) presented with bilateral congenital nuclear and cortical cataract, and no systemic anomalies. There was no family history of early onset cataract. He carried a heterozygous pathogenic de novo *GJA8* variant c.565C>T; p.(Pro189Ser).

### Family 13

A 36-year-old female (Family 13, I.2) presented with a history of bilateral congenital cataract, microcornea, glaucoma, nystagmus, and two missing teeth. She carried a heterozygous pathogenic *GJA8* variant c.592C>T; p.(Arg198Trp), also present in her two children (II.1 and II.2). Both II.1 and II.2 displayed bilateral cataract, microphthalmia, microcornea and nystagmus, and also exhibited extraocular features including mild microcephaly with speech delay (II.1), and mild microcephaly with mild motor delay (II.2). Additionally, individual II.2 presented with esotropia. All three affected individuals also carried a rare previously published heterozygous 545Kb duplication of 2q14.2 (Chr2:119320667-119865969 x3 [hg38]) of unknown significance [26].

### Family 14

A 17-year-old male (Family 14, II.1) presented with a history of bilateral congenital cataract and high myopia, but no systemic anomalies. He had a family history of high myopia, but not congenital cataract. He carried a heterozygous de novo 1.9Mb 1q21 microdeletion involving *GJA8* (Chr1:146020242-147909267 [hg38]) (Supplementary Fig. 1).

### Family 15

An 11-year-old female (Family 15, II.1) presented with bilateral childhood onset cataract, drusen in the left optic nerve at 7 years of age and bilateral central corneal lamellar opacities at age 10, but no systemic anomalies. She carried a heterozygous 4.4Mb 1q21 microdeletion involving *GJA8* (Chr1:145516559-149951620 [hg38]) (Supplementary Fig. 1). Parental DNA samples were unavailable for segregation analysis.

## Discussion

Here, we report variants affecting *GJA8* in 22 individuals from 15 new families, including 11 missense variants predicted pathogenic/likely pathogenic, one missense variant of uncertain significance, and two heterozygous 1q21 microdeletions of uncertain significance. Segregation analyses, available in ten families, demonstrated that six variants were de novo and four variants followed an autosomal dominant inheritance pattern. Of the 12 missense variants identified, one is novel [p.(Tyr66Asp)], another [p.(Thr39Met)] is very rare in public genomic databases and is not linked with any condition, while the remaining 10 have been previously reported in cases with eye anomalies. Together, our data expand the spectrum of *GJA8* variants, and help to delineate genotype-phenotype relationships.

Pathogenic variants in *GJA8* were initially described in individuals with isolated childhood cataract [10]. As a result, studies investigating this gene have mainly focused on single families or cohorts with congenital cataracts [15]. More recently, it emerged that individuals with *GJA8* variants can also display more severe lens anomalies (aphakia) or broader ocular phenotypes, including microphthalmia and anterior segment anomalies, typically in combination with cataract, and occasionally with extraocular features [11, 12, 16]. Our international study broadened the analysis to include subjects with a range of developmental eye anomalies. Six of the 13 families with missense *GJA8* variants identified herein presented with isolated cataract, without microphthalmia (families 1, 2, 3, 5, 11 and 12). Six families exhibited both cataract and microphthalmia (families 4, 6, 7, 8, 9 and 13), and one presented with microphthalmia and sclerocornea (family 10). Although it is not possible to draw conclusions about the relative frequency of isolated cataract versus cataract with microphthalmia in individuals with *GJA8* variants in this study, due to recruitment biases in the four cohorts, our screening confirms that alterations in this gene represent an important cause of microphthalmia. Interestingly, the proband in family 10 was diagnosed with microphthalmia and sclerocornea, but not cataract. While lens abnormalities can be ascertained using ultrasound in the presence of sclerocornea, this was not performed during the clinical assessment of family 10 and the proband was unavailable for further examination to determine the presence and/or characteristics of the lens. This illustrates how detailed phenotyping, including ultrasound, can potentially contribute to a better understanding of the range of features associated with *GJA8* variants and direct genetic testing. In addition to cataract, microphthalmia and sclerocornea, all affected individuals in families 3, 4, 5, 6, 8, 11 and 13 displayed other associated ocular features, including glaucoma, nystagmus, esotropia and amblyopia. However, many of these were secondary to the early onset cataract, structural eye anomalies and/or surgical interventions.

Of the 12 missense variants identified in this study, 10 have been previously reported in individuals with eye anomalies, allowing some genotype-phenotype correlations. The variants p.(Gly22Ser), p.(Val44Met), p.(Asp67Gly) and p.(Pro189Ser) were reported in individuals with cataract, but no structural eye anomalies, both in our cohort and previous studies [17, 27–32]. The variant p.(Gly94Arg), has been previously reported in four independent cases; the first presented with bilateral sclerocornea, microcornea and rudimentary lenses [12], the second with bilateral sclerocornea, microphthalmia, colobomas and congenital aphakia [11], the third with bilateral sclerocornea and congenital aphakia [13], and the fourth with microphthalmia and sclerocornea [33]. Here, we identified the same variant in a fifth case (family 10), also presenting with bilateral microphthalmia and sclerocornea. While the lens phenotype of the proband in family 10 was not assessed, this additional case strengthens the link between p.(Gly94Arg) and sclerocornea. Interestingly, sclerocornea occurring with aphakia/abnormal lens phenotype is also associated with biallelic variants in *FOXE3* [34]. Therefore, our findings indicate that genetic screening of individuals with sclerocornea and lens abnormalities, including aphakia, should involve *GJA8* in addition to *FOXE3*. Families 8 and 9 carry an alternative substitution affecting residue 94, p.(Gly94Glu). Interestingly, this change has been reported in a proband and mother with microphthalmia [14] and an individual with sclerocornea and microcornea [12], whereas families 8 and 9 in this study exhibited cataract and microphthalmia without sclerocornea. This demonstrates inter-familial phenotypic variability associated with this *GJA8* variant, with significant implications for clinical assessment and genetic counselling.

Such inter-familial phenotypic variability has been previously reported for other *GJA8* variants [11], and is observed for additional alterations in this study. For example, the probands carrying the variants p.(Arg76Cys) and p.(Pro88Leu) (families 6 and 7, respectively) exhibited congenital cataract and microphthalmia. However, previously reported individuals who carried these variants displayed isolated congenital cataracts [17, 35]. Furthermore, intra-familial phenotypic variation was also evident. In family 13, the affected mother and her two children carried the p.(Arg198Trp) variant and displayed cataract and microcornea features. However, her two children additionally manifested bilateral microphthalmia, microcephaly and developmental delay. The chromosome 2q14.2 duplication, previously reported in this family [26], is unlikely to explain the differences in phenotypes since all the affected individuals share this variant; no other variant was identified to explain the microcephaly. Interestingly, the same missense variant p.(Arg198Trp) was identified in a family with congenital cataract and microcornea, without any additional ocular or extraocular features [36], further highlighting inter-familial variability. Variation in phenotypic features is often attributed to environmental, genetic and local stochastic factors [37]. Six families with missense *GJA8* variants in the present study (families 6, 7, 9, 10, 11 and 13) displayed additional systemic anomalies, some explained by additional genetic variants. Of these, family 6 with tooth agenesis and family 11 with high myopia and congenital deafness, had additional variants in *WNT10A* and *COL11A1*, respectively, accounting for their extraocular features. Similarly, in the study by Ceroni et al. (2019), the systemic anomalies in some individuals with pathogenic *GJA8* variants were explicable by additional genetic variants [11]. Therefore, while no additional pathogenic variants were reported in the remaining individuals with systemic anomalies in the present study, further genetic analyses, especially of non-coding genomic regions, may be vital to unravel these additional features.

In addition to glycine 94, our study highlights two more mutational hotspots, threonine 39 and tyrosine 66. Two different alterations affecting threonine 39 have been reported, p.(Thr39Arg) and p.(Thr39Lys). The variant p.(Thr39Arg) has been described in two unrelated individuals, one with congenital cataract, microcornea and iris hypoplasia [38] and the other with congenital cataract, microphthalmia and corneal opacification [11]. The individual with p.(Thr39Lys) displayed microphthalmia, microcornea, cataract and anterior chamber anomalies with some systemic manifestations including neurodevelopmental delay, decreased body weight, and short stature (ClinVar: SCV004183581). We report a new third substitution of this residue, p.(Thr39Met), in an individual with isolated bilateral congenital cataract (family 2). This same variant is reported in one individual in the most recent release of gnomAD database (v4.1.0), but their ocular phenotype is not specified. Since this individual is from the UK Biobank, a population-based dataset that includes individuals who might have health conditions [39], it is possible that they have eye anomalies. Moreover, a recent study identified the same p.(Thr39Met) variant naturally occurring in cavefish with eye anomalies, including microphthalmia [40], providing further evidence that the variant may be causal. Similarly, we report a novel change of tyrosine 66, p.(Tyr66Asp), in an individual with bilateral cataract and microphthalmia (family 4). Three different pathogenic changes affecting this residue are reported in ClinVar [p.(Tyr66Cys) (SCV001379194), p.(Tyr66His) (SCV000952358) and p.(Tyr66Ser) (SCV001219479)], all in individuals with developmental cataract, without reported microphthalmia. Together, these data support the hypothesis that different substitutions at threonine 39 and tyrosine 66 can lead to developmental eye anomalies, predominantly cataract, with additional features perhaps influenced by the nature of the variant, but also other genetic and/or environmental factors.

Our study also identified two families with 1q21 microdeletions affecting *GJA8*. The impact of microdeletions affecting *GJA8* remains uncertain, due to several reported carriers having no apparent disease phenotype [11, 41, 42]. Interestingly, in one study, re-examination of a reportedly unaffected carrier of a 1q21 microdeletion revealed subtle lens opacities and a patent ductus arteriosus, which may be unrelated [43]. In contrast, in a family described by Ceroni et al. (2019) (family 15 [11]), in which the proband carried a heterozygous de novo 1q21 microdeletion involving *GJA8*, a pathogenic *FZD5* variant was subsequently identified [Individual 7 [42]], explaining their ocular phenotype. However, the proband who carried both the 1q21 microdeletion and *FZD5* variant displayed a more severe disease phenotype than her father and paternal cousin who only carried the *FZD5* variant. This suggests that the 1q21 microdeletions may either confer susceptibility to disease or contribute to its severity. Therefore, future studies of gene-gene interactions relevant to 1q21 microdeletions may unravel pathogenic mechanisms.

The expanding number of *GJA8* missense variants associated with severe ocular features contributes to our knowledge of the residues crucial for GJA8 activity. It was previously noted that cataract-associated *GJA8* variants clustered in the transmembrane domains, specifically between TM1 and TM2, while severe phenotypes, including microphthalmia, were more likely to be associated with alterations occurring in the extracellular and cytoplasmic loops [11, 44]. Our findings of cataract associated with the variants located in TM1 [p.(Gly22Ser) and p.(Thr39Met)], and microphthalmia and sclerocornea associated with variants in ICL [p.(Gly94Glu) and p.(Gly94Arg)] support this view. However, in our cohorts, p.(Arg76Cys) and p.(Pro88Leu), both located in TM2, were also found to be associated with severe ocular phenotypes including microphthalmia (families 6 and 7). This suggests that the phenotype associated with *GJA8* variants might not be domain-specific, but rather dependent on the particular residue and/or substitution.

The complex ocular phenotypes displayed by individuals with *GJA8* variants could also partly be explained by the expression pattern of *GJA8* within the eye. *GJA8* is expressed widely throughout the lens [8], and also in the cornea [45, 46], indicating an additional role in corneal gap junctions. A related connexin, *GJA3* (Cx46), is predominantly expressed in lens fibres, and individuals with *GJA3* variants display cataract-only phenotypes. These differences are recapitulated by *Gja3* and *Gja8* mouse models where *Gja3*-null mice had cataract but normal eye size [47], while *Gja8*-null mice developed cataracts earlier and exhibited smaller lenses and microphthalmia [5, 6]. Mice/humans with variants in other cataract-associated genes, such as crystallins can also manifest with complex features including corneal anomalies, iris hypoplasia and microphthalmia [(*CRYAA* [48]; *CRYBB1* [49]; *CRYBA4* [50]]. This further indicates that several cataract-associated genes, including *GJA8*, may have broader roles in ocular growth and development.

In conclusion, we report 22 individuals from 15 new families with *GJA8* variants identified from a large international multicentre cohort of individuals with developmental eye anomalies. We highlight that *GJA8* variants represent an important source of genetic diagnoses not only for individuals with early onset cataract, but also for individuals with developmental eye anomalies, including microphthalmia and sclerocornea. Our data illustrates *GJA8* mutational hotspots, and significant inter- and intra-familial variation associated with *GJA8* variants. Further genotype-phenotype studies of individuals with rare developmental eye anomalies will be vital in defining the role of *GJA8* and other genes in their pathogenesis. This, in turn, will improve diagnosis, counselling of families, and provide insight into future therapies.

## Supplementary information

Supplementary Information

## Data Availability

Variants were submitted to ClinVar database with the following accession numbers: SCV005044863, SCV005044864, SCV005044865, SCV005044866, SCV005044867, SCV005044868, SCV005044869, SCV005044870, SCV005044871, SCV005044872, SCV005044873, SCV005044874, SCV005044875, SCV005044876.
