# Supplementary material for: GJA8-associated developmental eye disorders: a new multicentre study highlights mutational hotspots and genotype-phenotype correlations
Source: Eur J Hum Genet. 2025 Apr 30;33(7):860–9. doi: 10.1038/s41431-025-01843-8 (PMC12229616; doi:10.1038/s41431-025-01843-8)
Supplement: Supplementary file 1 — Supplementary Information [file 41431_2025_1843_MOESM1_ESM.pdf]

***GJA8*-associated developmental eye disorders: a new multicentre study highlights mutational hotspots and genotype-phenotype correlations**

Supplementary Methods

Variant filtering protocol applied to each cohort:

UK cohort

Whole Exome/Genome Sequencing (WES/WGS) data were aligned to the human genome using the Isaac Genome Alignment Software. Variants were identified using Isaac Variant Caller, annotated with ANNOVAR (1) and filtered using a customised in-house pipeline prioritising variants with a functional effect on coding or splicing regions and a minor allele frequency (MAF) ≤0.5% in gnomAD v4.1.0. Annotations included multiple *in silico* predictions and conservation scores obtained from dbNSFP v4.7. Variants in genes included in a UK diagnostic gene panel for structural eye disorders (v4.0) (https://panelapp.genomicsengland.co.uk/panels/509/) were prioritised.

French cohort

Variants were filtered based on data quality (depth >30X) and variant frequency in control population databases, including gnomAD. Variants identified >4 times in the same run of 16 unrelated patients were excluded as artefacts. All variants were classified manually based on ACMG/AMP guidelines (2).

Spanish cohort

Clinical exome sequencing

Clinical exome sequencing was performed using the Clinical Exome Solution (CES), a gene panel developed by Sophia Genetics (Boston, MA, United States). Libraries were prepared following the manufacturer's instructions and sequenced on a NextSeq500 platform (Illumina) (3) and data analysed using the commercial platform SOPHiA DDM (Sophia Genetics).

Custom panels and WES

Variant annotation was performed using an in-house pipeline, NextVariantFJD, available at https://github.com/TBLabFJD/NextVariantFJD. This includes the variant effect predictor (VEP) (4) for SNVs and indels and AnnotSV (5) for SVs, plus additional custom information.

Variants identified through WES were also prioritised using GLOWgenes (www.glowgenes.org), a network-based algorithm designed to highlight new candidate genes for rare disorders (6).

Copy Number Variants (CNVs) were detected using four different algorithms based on read depth: CoNVaDING (7), ExomeDepth (8), CODEX2 (9) and panelcnMOPS (10).

USA cohort

Exome data was annotated according to human genome build GRCh37/hg19 and filtered using VarSeq software (Golden Helix; Bozeman, MT) for novel/ultra-rare variants in *GJA8* based on gnomAD data (11). *In silico* tools, including CADD (12) and AlphaMissense (13), were used to predict the functional effects of variants and their corresponding amino acid substitutions. Variant validation and segregation analyses were performed using Sanger sequencing. Variants were classified according to the ACMG/AMP guidelines (2). CNVs were detected using CNV Caller in VarSEq (WES data) and/or microarray-based comparative genomic hybridization (aCGH).

Supplementary Figure 1

Copy number variants involving *GJA8*. A schematic from UCSC Genome Browser (NCBI Genome Build GRCh38/hg38). Top track shows multiple regions with segmental duplications. Middle track contains genomic locations of 1q21 microdeletions involving *GJA8*; CNVs identified in the present study and those previously reported in an AMC cohort (14) are represented by red bars, with family identifiers adjacent. RefSeq MANE select transcripts, being reference isoforms representative of genes with multiple isoforms, are indicated in dark blue on the lowest track. The vertical blue line highlights the location of *GJA8* in the genomic region displayed. *Family with additional pathogenic *FZD5* variant reported by Holt *et al.* (15).

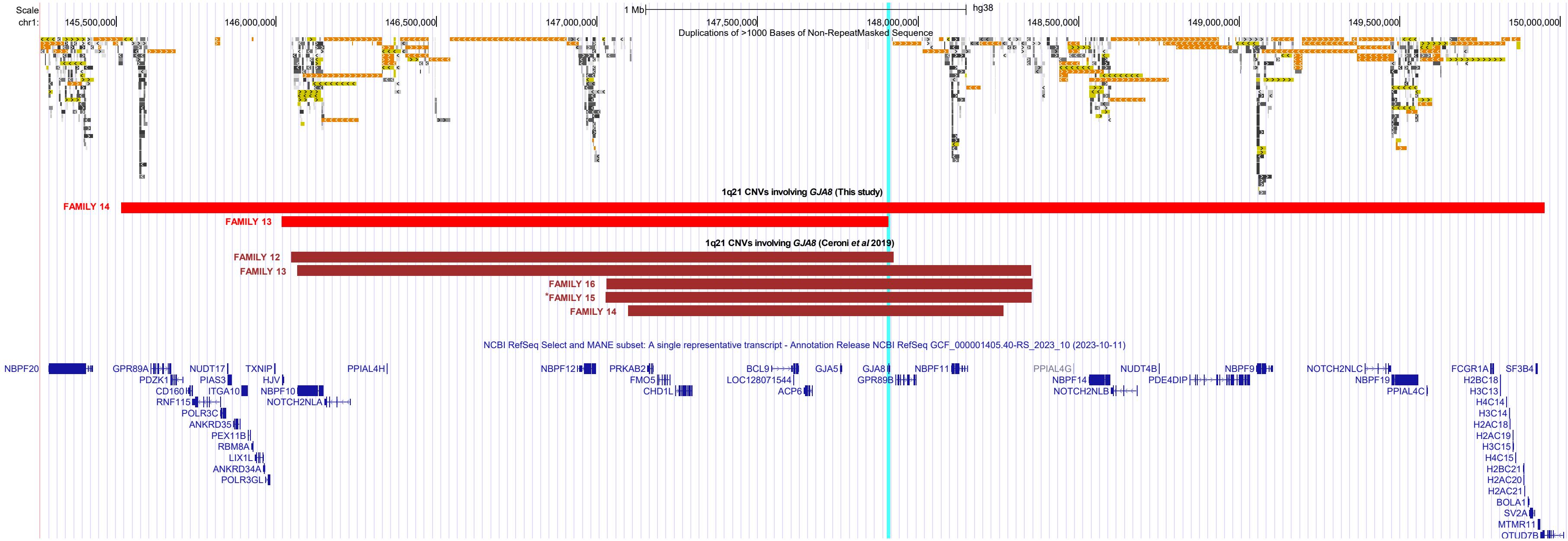

Supplementary References

1. Wang K, Li M, Hakonarson H. ANNOVAR: functional annotation of genetic variants from high-throughput sequencing data. Nucleic Acids Res. 2010;38(16):e164.
2. Richards S, Aziz N, Bale S, Bick D, Das S, Gastier-Foster J, et al. Standards and guidelines for the interpretation of sequence variants: a joint consensus recommendation of the American College of Medical Genetics and Genomics and the Association for Molecular Pathology. Genet Med. 2015;17(5):405-24.
3. Martin-Merida I, Avila-Fernandez A, Del Pozo-Valero M, Blanco-Kelly F, Zurita O, Perez-Carro R, et al. Genomic Landscape of Sporadic Retinitis Pigmentosa: Findings from 877 Spanish Cases. Ophthalmology. 2019;126(8):1181-8.
4. McLaren W, Gil L, Hunt SE, Riat HS, Ritchie GR, Thormann A, et al. The Ensembl Variant Effect Predictor. Genome Biol. 2016;17(1):122.
5. Geoffroy V, Herenger Y, Kress A, Stoetzel C, Piton A, Dollfus H, et al. AnnotSV: an integrated tool for structural variations annotation. Bioinformatics. 2018;34(20):3572-4.
6. de la Fuente L, Del Pozo-Valero M, Perea-Romero I, Blanco-Kelly F, Fernández-Caballero L, Cortón M, et al. Prioritization of New Candidate Genes for Rare Genetic Diseases by a Disease-Aware Evaluation of Heterogeneous Molecular Networks. Int J Mol Sci. 2023;24(2).
7. Johansson LF, van Dijk F, de Boer EN, van Dijk-Bos KK, Jongbloed JD, van der Hout AH, et al. CoNVaDING: Single Exon Variation Detection in Targeted NGS Data. Hum Mutat. 2016;37(5):457-64.
8. Plagnol V, Curtis J, Epstein M, Mok KY, Stebbings E, Grigoriadou S, et al. A robust model for read count data in exome sequencing experiments and implications for copy number variant calling. Bioinformatics. 2012;28(21):2747-54.
9. Jiang Y, Wang R, Urrutia E, Anastopoulos IN, Nathanson KL, Zhang NR. CODEX2: full-spectrum copy number variation detection by high-throughput DNA sequencing. Genome Biol. 2018;19(1):202.
10. Povysil G, Tzika A, Vogt J, Haunschmid V, Messiaen L, Zschocke J, et al. panelcn.MOPS: Copy-number detection in targeted NGS panel data for clinical diagnostics. Hum Mutat. 2017;38(7):889-97.
11. Chen S, Francioli LC, Goodrich JK, Collins RL, Kanai M, Wang Q, et al. A genomic mutational constraint map using variation in 76,156 human genomes. Nature. 2024;625(7993):92-100.
12. Kircher M, Witten DM, Jain P, O'Roak BJ, Cooper GM, Shendure J. A general framework for estimating the relative pathogenicity of human genetic variants. Nat Genet. 2014;46(3):310-5.
13. Cheng J, Novati G, Pan J, Bycroft C, Žemgulytė A, Applebaum T, et al. Accurate proteome-wide missense variant effect prediction with AlphaMissense. Science. 2023;381(6664):eadg7492.
14. Ceroni F, Aguilera-Garcia D, Chassaing N, Bax DA, Blanco-Kelly F, Ramos P, et al. New GJA8 variants and phenotypes highlight its critical role in a broad spectrum of eye anomalies. Hum Genet. 2019;138(8-9):1027-42.
15. Holt R, Goudie D, Verde AD, Gardham A, Ramond F, Putoux A, et al. Individuals with heterozygous variants in the Wnt-signalling pathway gene *FZD5* delineate a phenotype characterized by isolated coloboma and variable expressivity. Ophthalmic Genet. 2022;43(6):809-16.
